# Ocoxin Modulates Cancer Stem Cells and M2 Macrophage Polarization in Glioblastoma

**DOI:** 10.1155/2019/9719730

**Published:** 2019-08-05

**Authors:** Esther Hernández-SanMiguel, Ricardo Gargini, Teresa Cejalvo, Berta Segura-Collar, Paula Núñez-Hervada, Rafael Hortigüela, Juan M. Sepúlveda-Sánchez, Aurelio Hernández-Laín, Angel Pérez-Núñez, Eduardo Sanz, Pilar Sánchez-Gómez

**Affiliations:** ^1^Neurooncology Unit, Instituto de Salud Carlos III-UFIEC, Madrid, Spain; ^2^Centro de Biología Molecular, CSIC, Madrid, Spain; ^3^Unidad Multidisciplinar de Neurooncología, Hospital Universitario 12 de Octubre, Madrid, Spain; ^4^Catalysis S.L., Madrid, Spain

## Abstract

Glioblastoma (GBM) is the most common and devastating primary brain tumor. The presence of cancer stem cells (CSCs) has been linked to their therapy resistance. Molecular and cellular components of the tumor microenvironment also play a fundamental role in the aggressiveness of these tumors. In particular, high levels of hypoxia and reactive oxygen species participate in several aspects of GBM biology. Moreover, GBM contains a large number of macrophages, which normally behave as immunosuppressive tumor-supportive cells. In fact, the presence of both, hypoxia and M2-like macrophages, correlates with malignancy and poor prognosis in gliomas. Antioxidant agents, as nutritional supplements, might have antitumor activity. Ocoxin® oral solution (OOS), in particular, has anti-inflammatory and antioxidant properties, as well as antitumor properties in several neoplasia, without known side effects. Here, we describe how OOS affects stem cell properties in certain GBMs, slowing down their tumor growth. In parallel, OOS has a direct effect on macrophage polarization *in vitro* and *in vivo*, inhibiting the protumoral features of M2 macrophages. Therefore, OOS could be a feasible candidate to be used in combination therapies during GBM treatment because it can target the highly resilient CSCs as well as their supportive immune microenvironment, without adding toxicity to conventional treatments.

## 1. Introduction

Glioblastomas (GBMs) are the most aggressive form of primary brain tumors. Histologically, they are characterized by pronounced hypercellularity, aberrant vasculature, and necrotic regions [1]. Standard treatment of GBM consists of maximal safe surgical resection followed by focal, fractionated radiotherapy, in combination with concurrent and adjuvant chemotherapy with the DNA alkylating agent temozolomide (TMZ) [2]. In any case, the prognosis is still grim, with less than 10% of GBM patients surviving 5 years after diagnosis, so novel treatment modalities are urgently required.

GBM cancer stem cells (CSCs) are able to self-renew, differentiate and repopulate the whole bulk of the tumor, and they have been associated with tumor relapse after treatment [3]. Maintenance of an undifferentiated state of GBM CSCs seems to be controlled by cues from their niches, mainly vascularized areas and hypoxic regions [4, 5]. Paradoxically, hypoxia is associated with an increase in reactive oxygen species (ROS) [6]. In fact, molecules that induce a decrease in endogenous ROS levels have been recently associated with the inhibition of CSC-like properties in GBM [7, 8].

Dietary supplements (including antioxidants) are widely used among patients with cancer, with the potential to be anticancer and antitoxic agents, reducing side effects. Whereas some authors have reported undesirable interactions with conventional therapies, others have suggested a synergistic effect [9, 10]. Ocoxin® oral solution (OOS) is a nutritional supplement with recognized antioxidant, anti-inflammatory and immunomodulatory properties. The solution is composed, among others, of green tea extract, glyzyrrhicic acid, and vitamins C, B6 and B12, which have undergone a molecular activation process that boosts their antioxidant and biological activities. It was synthesized by combining two products, Viusid® and Ocoxin®. The Viusid component has shown beneficial effects in patients suffering from chronic hepatitis C and cirrhosis, showing antioxidant and immunomodulatory effects [11, 12]. OOS has also been tested in several cancer clinical trials, resulting in a significant improvement in the quality of life of patients, better tolerance to conventional therapies, and an increase in the survival index [13] (NCT01392131). Moreover, OOS antitumor effects have been validated *in vitro* and *in vivo* in preclinical breast cancer [14] and acute myeloid leukemia models [15], playing an inhibitory role for the liver metastasis of colorectal carcinoma [16, 17]. In all cases, when administered to animal models, OOS showed no detrimental effects. In fact, it even improved the overall health state of the mice.

Here, we have tested the effect of OOS in GBM models. For that we have used primary cell lines, derived from patient samples. We have observed that OOS has a dual function, it reduces tumor growth in some GBM through the modulation of CSC properties, and it shows a striking capacity to inhibit M2-like macrophage polarization, both *in vitro* and in the tumor environment. Moreover, pretreatment with OOS reduces the protumoral function of M2 macrophages. Therefore, OOS could be a good candidate to be used in combination therapies during GBM treatment, especially if we consider that it has been marketed for years without any report of adverse effects.

## 2. Materials and Methods

### 2.1. GBM Cell Culture

GBM1 (L0627) was provided by Rosella Galli (San Raffaele Scientific Institute, Milan, Italy); GBM2 (12O15) and GBM3 (12O01) were obtained by dissociation of human GBM surgical specimens from patients treated at Hospital Universitario 12 de Octubre (Madrid, Spain). We obtained an informed consent for study participation from both patients. None of them was under the age of 18. The study was performed with the approval and following the guidelines of the Research Ethics Committee of Hospital 12 de Octubre (CEI 14/023). Primary GBM cells were cultured in Neurobasal medium (Invitrogen) supplemented with B27 (1 : 50, Invitrogen), Glutamax (1 : 100, Invitrogen), penicillin-streptomycin (1 : 100, Lonza), 0.4% heparin (Sigma-Aldrich), 40 ng/ml EGF (PeproTech), and 20 ng/ml bFGF_2_ (PeproTech) and passaged after enzymatic disaggregation using Accumax (Millipore), as previously described [18].

### 2.2. GBM *In Vitro* Assays

For viability assays, 5000 cells were seeded in triplicate wells of a 96-multiwell plate coated with Matrigel (Becton-Dickinson, 15 mg/ml stock solution diluted 1 : 100 in DMEM medium (Lonza)). 24 h later, cells were treated with OOS (1 : 100) (Catalysis S.L.) and viability was measured after 72 h of treatment. For that, cells were incubated with Hoechst 33342 (1 : 200, Sigma-Aldrich) and propidium iodide (1 : 1000, Merck) and fluorescence was measured in a Cytell Cell Imaging System (GE Healthcare Life Sciences). Wells containing nontreated cells were considered as 100% viability for each tested cell line. For self-renewal assays, GBM neurospheres were disaggregated into single cells and plated in fresh medium in the absence of OOS at a clonal density of 2.5 cells/*μ*l in triplicate wells of a 96-multiwell plate. 24 hours later, GBM cell lines were treated with OOS (1 : 100). The percentage of self-renewing cells was determined 6 days after treatment by counting the number of individual neurospheres that originated from plated cells. Nontreated cells were used as a control.

### 2.3. Macrophage Isolation and Culture

Peritoneal macrophages were isolated as described in [19]. In brief, for macrophage isolation, 2.5 ml of 3% thioglycollate (Sigma-Aldrich) was injected into the peritoneum of C57BL/6 mice. Four days later, peritoneal cells were harvested and 15000 cells/well were seeded in a 96-multiwell plate for viability and ROS assays. Otherwise, 2.5 × 10^6^ cells/well were seeded in a 6-multiwell plate for RNA isolation. Cells were cultured in RPMI 1640 (Fisher Scientific) with 10% of heat-inactivated fetal bovine serum (FBS, Fisher Scientific) and penicillin-streptomycin (1 : 100, Lonza) for 3 h. The amount of FBS was then reduced to 2% for overnight cell starvation. After overnight starvation, cells were treated with or without 1 : 100 OOS, in the presence of the differentiation inducers: LPS (200 ng/ml, InvivoGen), IL4 (20 ng/ml, PeproTech) or GBM1 conditioned medium (CM, 1 : 1) (obtained after 72 h incubation of GBM1 cells in neurosphere media). Control cells were maintained in 2% FBS medium. After 24 h, LPS and IL4 were removed but OOS was maintained for another 24 h before macrophage analysis.

### 2.4. Macrophage *In Vitro* Assays

Macrophage viability was assessed using AlamarBlue reagent (Fisher Scientific). The reagent was added to the media (1 : 10) and incubated for 4 h at 37°C and 5% CO_2_, protected from light. Fluorescence of the samples was measured (excitation at 560 nm and emission at 600 nm) in an Infinite 200 PRO microplate reader (Tecan). Wells containing only culture media were used as a background control for all the samples measured, and wells containing nontreated cells were considered as 100% viability. ROS measurement was performed using dihydroethidium (DHE) reagent (Cayman Chemical). DHE was added to the media (1 : 800) and incubated for 1 h at 37°C and 5% CO_2_, protected from light. Fluorescence of the samples was measured in a Cytell Cell Imaging System. Wells containing only culture media were used as a background control for all the samples measured, and wells containing nontreated cells were considered as 100% ROS. For the coculture experiment, 2.5 × 10^6^ macrophages were seeded per 6-multiwell plate. After the overnight starvation, cells were treated with or without 1 : 100 OOS, in the presence of IL4 (20 ng/ml, PeproTech). Control macrophages were maintained in 2% FBS medium. After 24 h, IL4 was removed but OOS was maintained for another 24 h. After OOS removal, macrophages were washed and freshly dissociated GBM1 cells (expressing the luciferase reporter) (250000 cells) were added on top of the macrophages (in GBM media without growth factors). Three days later, luciferin was added (150 *μ*g/ml) and luminiscence was measured in an IVIS equipment (Perkin Elmer).

### 2.5. Mouse Xenograft Assays

Animal care and experimental procedures were performed in accordance with the European Union and National Guidelines for the use of animals in research and were reviewed and approved by the Research Ethics and Animal Welfare Committee at our institution (Instituto de Salud Carlos III, Madrid) (PROEX 244/14). Heterotopic and orthotopic xenografts were performed as previously described [20]. For heterotopic xenografts, 1 × 10^6^ cells were resuspended in culture media with Matrigel (1 : 10, BD) and then subcutaneously injected into athymic nude *Foxn1^nu^* mice (Harlan Iberica). When the subcutaneous tumors were noticeable (around 4 mm in diameter), OOS or water was orally administered to the mice (100 *μ*l/day, 5 days/week). During the treatments, tumors were measured with a caliper twice a week until mice sacrifice. Tumor volume was calculated as 1/2 (length × width^2^). Relative tumor growth was calculated in relation to tumor volume at day 1 of treatment. For orthotopic xenografts, stereotactically guided intracranial injections in athymic nude *Foxn1^nu^* mice were performed by administering 0.5 × 10^5^ GBM1 cells resuspended in 2 *μ*l of culture media. The injections were made into the striatum (coordinates: A-P, –0.5 mm; M-L, +2 mm; and D-V, –3 mm; related to the bregma) using a Hamilton syringe. Mice were orally treated with OOS or water (200 *μ*l/day, 5 days/week) 3 weeks after the intracranial injections until mice were sacrificed at the onset of symptoms.

### 2.6. Tumor Tissue Analysis

At the endpoint, subcutaneous tumors or brain tumors were dissected and the tissue was fresh frozen for molecular analysis, dissociated for flow cytometry analysis or fixed o/n in 4% paraformaldehyde (Merck) and embedded in paraffin. Paraffin-embedded tissue was cut with a microtome (Leica Microsystems) (3 *μ*m sections), and sections were stained with hematoxylin-eosin (H&E) or incubated with specific antibodies for immunohistochemical- (IHC-) DAB staining.

### 2.7. Western Blot (WB)

For immunoblot analysis, cells were collected and rinsed in cold PBS. Samples were resuspended in 0.1% SDS-RIPA buffer supplemented with a protease inhibitor cocktail (Roche), incubated 20 minutes on ice and centrifuged at 14000 rpm for 15 min at 4°C. Protein concentration was determined using a commercially available colorimetric assay (Pierce BCA Protein Assay Kit, Thermo Scientific). Approximately 20 *μ*g of protein were resolved by 12% SDS-PAGE and transferred onto a nitrocellulose membrane. Membranes were blocked for 1 h at room temperature in 5% BSA in TBS-T (10 mM Tris-HCl, pH 7.5, 100 mM NaCl and 0.1% Tween-20) and then incubated o/n at 4°C with the corresponding primary antibody diluted in 5% BSA in TBS-T. After washing 3 times with TBS-T, membranes were incubated for 1 hour at room temperature with their corresponding secondary antibody diluted in TBS-T. Detection was done by enhanced chemiluminescence with ECL (Millipore). Primary and secondary antibodies are shown in Supp.  Table S1 and S2, respectively.

### 2.8. Quantitative Real-Time PCR (qRT-PCR)

RNA was extracted from both frozen pellets of cells or frozen tissue sections with the High Pure RNA Isolation Kit (Roche) following the manufacturer's instructions. Total RNA (1 *μ*g) was reverse transcribed with the PrimeScript RT Reagent Kit (TAKARA) in a total volume of 20 *μ*l. The product of this retrotranscription was tenfold diluted for quantitative PCR analysis. Quantitative real-time PCR (qRT-PCR) was performed using the Light Cycler 480 (Roche) with SYBR Premix Ex Taq (TAKARA) in LightCycler® 480 Multiwell Plates using 10 *μ*M of forward and reverse primers and 2 *μ*l of cDNA template (tenfold diluted). Cycling conditions included an initial denaturation for 10 minutes at 95°C, followed by 45 cycles of 10 s at 95°C, 10 s at primer hybridization temperature and 10 s at 72°C. Quantification of gene expression was performed by the delta-delta Ct method, and Ct values were calculated following the manufacturer's instructions (LightCycler Software, Roche). The expression of the housekeeping genes *Actin* or *RPII* was used as an internal expression control. Primers used are indicated in Supp.  Table S3.

### 2.9. Flow Cytometry Analysis

Tumor cells were disaggregated into individual cells with Accumax (5 min, RT) and erythrocytes were lysed with Quicklysis (15 min, RT; Cytognos) before staining. Cells were stained with anti-CD44-FITC (ImmunoTools, Supplementary Table S1) diluted in PBS+0.5% BSA+2 mM EDTA (staining buffer) for 20 min on ice and treated with PI (5 *μ*g/ml, Sigma-Aldrich) for 5 min on ice. After staining, cells were washed with staining buffer and analyzed by flow cytometry (FACSCalibur, Becton Dickinson) using the FlowJo software.

### 2.10. Immunohistochemical Analysis (IHC)

Tumors were fixed o/n at 4°C in 4% paraformaldehyde (PFA, Merck), rinsed with 0.1 M phosphate buffer (PB), and dehydrated by an ethanol gradient before embedding in paraffin for microtome sectioning. Tumors were cut with a microtome (Leica Microsystems), and paraffin sections (3 *μ*m) were dewaxed and rehydrated. Antigen retrieval was achieved by microwaving the sections in 10 mM sodium citrate (pH 6.0) and endogenous peroxidase was blocked with 0.3% hydrogen peroxide. Tissue sections were blocked with 5% BSA+10% FBS in PB-Triton X-100 (0.1%) and incubated o/n at 4°C with anti-Activated Caspase 3 in blocking buffer. Sections were incubated with a secondary antibody labelled with biotin (2 hours at room temperature) before incubation with ABC-Peroxidase Solution (Thermo Scientific) for 30 min at room temperature. The peroxidase activity was developed with DAB (Vector) and sections were counterstained with hematoxylin. Images were acquired with a Leica DM4B microscope and analyzed by using ImageJ software. The primary and secondary antibodies used are indicated in Supp.  Table S1 and S2, respectively.

### 2.11. Statistical Analysis

The survival of nude mice was analyzed by the Kaplan-Meier method and evaluated with a two-sided log-rank test. Student's *t*-test was performed for statistical analysis of *in vitro* studies. Data in graphs are presented as means ± SEM. ^∗^ *P* ≤ 0.05; ^∗∗^ *P* ≤ 0.01; ^∗∗∗^ *P* ≤ 0.001. Statistical values of *P* > 0.05 were not considered significant.

## 3. Results

### 3.1. OOS Inhibits the Self-Renewal Capacity of Some GBM CSCs

**Figure 1:**
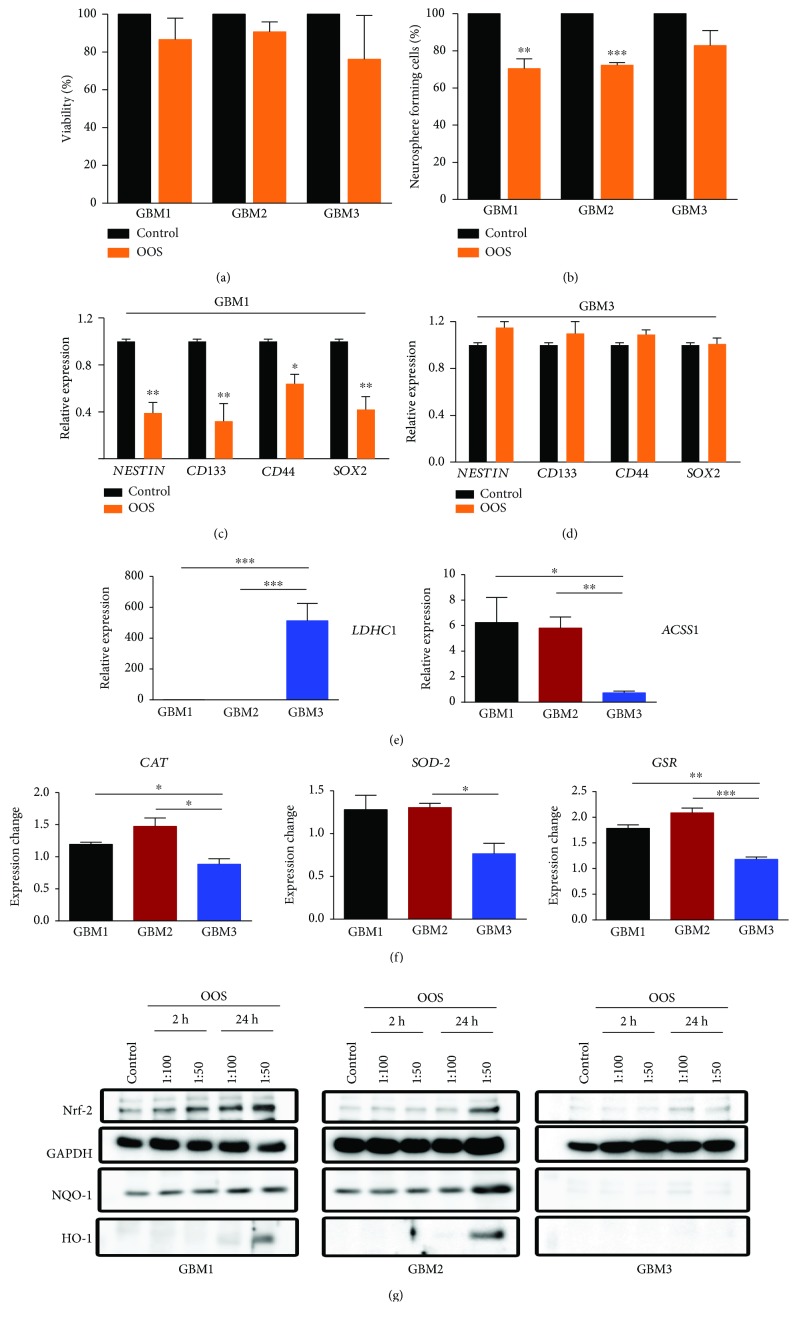
OOS inhibits the self-renewal capacity of GBM cells. (a) Cell viability of GBM cells in response to OOS (3d), *n* = 3. (b) Formation of GBM clonal spheres in the presence of OOS (6d), *n* = 3. (c, d) Relative expression (qRT-PCR) of CSC-related markers in GBM1 (c) and GBM3 (d) cells treated in the absence or in the presence of OOS (24 h), *n* = 3. (e) Relative expression (qRT-PCR) of metabolic markers in the three different GBM lines, *n* = 3. (f) Induction of the expression of several antioxidant enzymes in the presence of OOS (24 h), *n* = 3. *RPII* was used for normalization in all qRT-PCRs. (g) GBM cells were treated with OOS (1 : 100 or 1 : 50) for 2 or 24 h and expression of Nrf-2, NQO-1, and HO-1 was measured by WB. Nontreated spheres were used as a control and the expression of GAPDH as a loading control. The blots in the image were cropped from the same gel (full-length blots are shown in Supp. Figure S1). ^∗^ *P* ≤ 0.05; ^∗∗^ *P* ≤ 0.01; ^∗∗∗^ *P* ≤ 0.001.

To explore the antitumor activity of OOS we used three different primary GBM cell lines derived from human samples, grown in the absence of serum and in the presence of growth factors, in the form of floating neurospheres enriched in CSCs [21]. Three days' incubation in the presence of OOS (1 : 100) did not produce a significant change in cell viability (Figure 1(a)). However, the same concentration of OOS inhibited the capacity of GBM1 and GBM2 cells to grow at highly diluted conditions (self-renewal assay) (Figure 1(b)), which is a feature related to CSCs and tumor-initiating properties [22]. Interestingly, we did not observe a significant effect of OOS on the self-renewal capacity of GBM3 cells. In agreement with these results, we observed a significant inhibition of CSC-related markers in GBM1 cells treated with OOS for 24 h (Figure 1(c)), whereas the supplement did not change the expression of these genes in GBM3 cells (Figure 1(d)).

GBM is a very heterogeneous group of tumors. In fact, we know that the behavior of GBM1 and GBM2 differs from GBM3 as the first two grow in a highly angiogenic manner in the mouse's brain, whereas the third one generates very invasive tumors (Gargini et al., manuscript in preparation) [23]. We have also observed differences in the metabolic profile of GBM3 cells, in comparison with GBM1 and GBM2 cells, with a clear upregulation of glycolytic enzyme *LDHC1* (Lactate-dehydrogenase-C1) expression and a significant reduction in the expression of the mitochondrial enzyme *ACSS1* (Acyl-CoA synthetase short-chain family-member1) (Figure 1(e)). Moreover, OOS induced the expression of several antioxidant enzymes in GBM1 and GBM2, but not in GBM3 (Figure 1(f)). We also checked the status of the detoxifying Nrf-2 (Nuclear factor erythroid-2-related factor 2) system. Nrf-2 can be modulated in response to redox imbalance [24] and it induces the expression of several antioxidative genes like hemoxygenase-1 (HO-1) and NAD(P)H Quinone Dehydrogenase 1 (NQO-1) [25]. OOS augmented the levels of Nrf-2 protein in the three cell lines, although the expression of its targets was induced in GBM1 and GBM2 cells but not in the GBM3 line, being GBM1 the one that responds to lower levels of OOS at shorter times (Figure 1(g)). All these results reinforce the idea that the metabolic and redox profiles of GBM3 cells, compared to GBM1 and GBM2 cells, are quite different and this might be the cause for their lack of response to OOS. Moreover, the results suggest that the expression of some of these proteins could be used as biomarkers of response to OOS.

### 3.2. The Growth of Some GBMs Is Impaired by Systemic Administration of OOS

**Figure 2:**
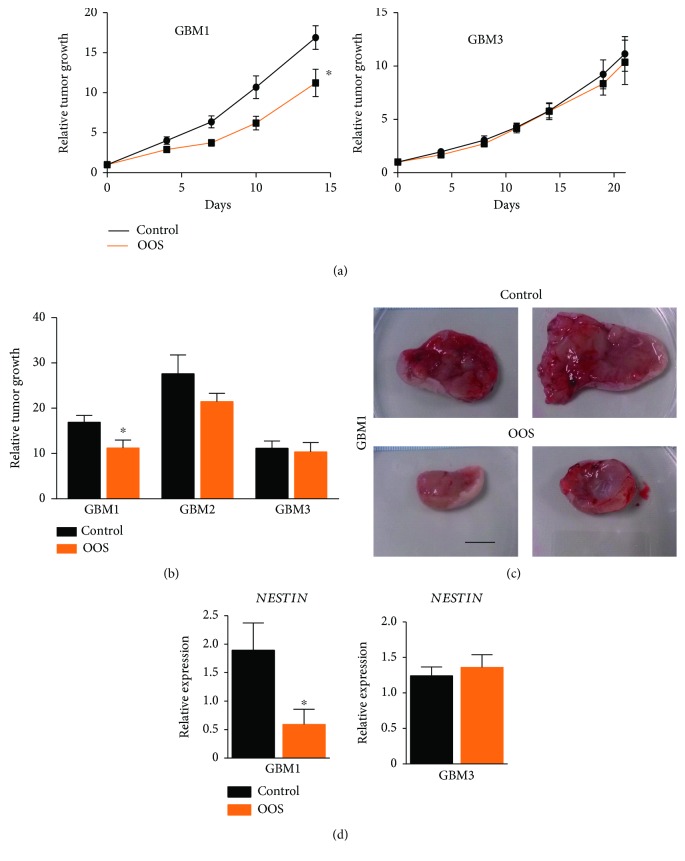
OOS inhibits GBM growth. GBM1-3 cells were injected subcutaneously into the flanks of nude mice; when tumors became visible, animals started receiving daily oral doses of OOS (100 *μ*l/day, 5 days/week) or water (control). Relative tumor growth at different time points (a) or the time of sacrifice (b) is represented, *n* = 8. (c) Representative pictures of the tumors are shown. (d) *NESTIN* expression (qRT-PCR) in flank tumors after OOS treatment, *n* = 4. *RPII* was used for normalization in all qRT-PCRs. ^∗^ *P* ≤ 0.05. Scale bar = 1 cm.

CSCs in GBM have been associated with tumor initiation and growth. In order to test if the inhibition of self-renewal induced by OOS *in vitro* has an effect on tumor growth, we injected the three primary GBM lines into the flanks of immunodeficient (nude) mice. When tumors became visible, animals received intragastric administration of OOS and tumor growth was monitored with a caliper. The results showed that there was a significant reduction in tumor growth in GBM1, a small but not significant inhibition of GBM2 growth, and no reduction in GBM3 tumors (Figures 2(a)–2(c)). Histological analysis of GBM1 tumors showed no changes in the number of mitoses or in the number of Activated Caspase 3-positive cells (Supp.  Figure S2), suggesting that OOS does not affect overall tumor growth or survival, which correlates with the lack of effect on cell viability (Figure 1(a)). Moreover, we observed a significan change in GBM1 tumors in the expression of the marker *NESTIN*, associated with GBM CSCs, which was not observed in GBM3 tumors (Figure 2(d)). This reinforces the idea that OOS reduces the stem cell properties of certain GBMs, affecting tumor growth.

**Figure 3:**
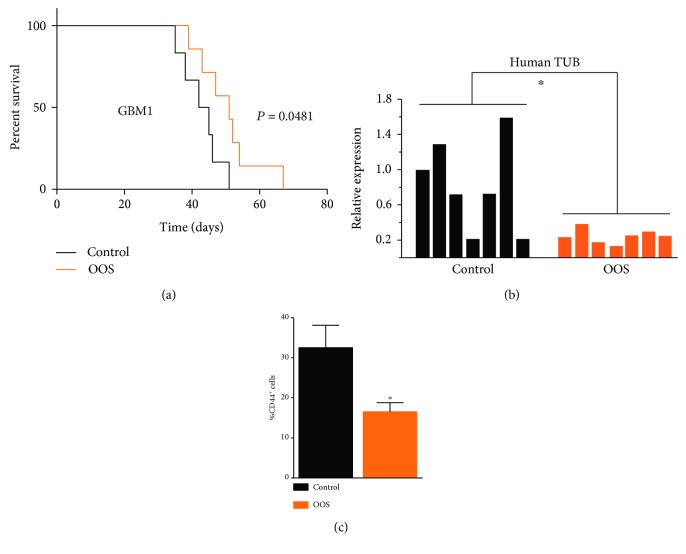
OOS inhibits intracranial tumor growth. GBM1 cells were injected into the brains of nude mice. The animals were treated orally with daily doses of OOS (200 *μ*l, 5 days/week) or water (control). (a) Animal survival was evaluated using a Kaplan-Meier survival curve and the differences in survival times were analyzed with a log-rank test, *n* = 7. (b) qRT-PCR analysis of *human β-tubulin* (*human TUB*) related to *RPII* (human and mouse) levels in the dissected brains. Each bar represents one tumor. (c) Percentage of CD44^+^ cells in dissociated brain tumors analyzed by flow cytometry, *n* = 3. ^∗^ *P* ≤ 0.05.

With the goal of measuring the effect of OOS in an orthotopic setting, we performed an intracranial injection of GBM1 cells in nude mouse brains. We observed that systemic administration of OOS reduces tumor burden (Figure 3(a)). To further quantify tumor growth, we dissected the right hemispheres from the mouse brains and we measured the expression of *β-tubulin*, with primers that recognize specifically the human sequence. We compared it with the expression of the *RNA polymerase subunit-2* (*RPII*), measured with primers that equally recognize both human and mouse sequences. In the dissected brains, we observed a significant reduction in the human component (Figure 3(b)), confirming that the effects of OOS are visible in the brain. We also dissociated the injected hemispheres and we analyzed the cells using flow cytometry, measuring a strong reduction in the percentage of CD44^+^ cells (Figure 3(c)). CD44 is considered a marker for GBM CSCs [26, 27], reinforcing the idea that OOS affects GBM1 tumor growth by impairing CSCs.

### 3.3. OOS Stimulates Changes in the Macrophage Component of GBM Tumors

**Figure 4:**
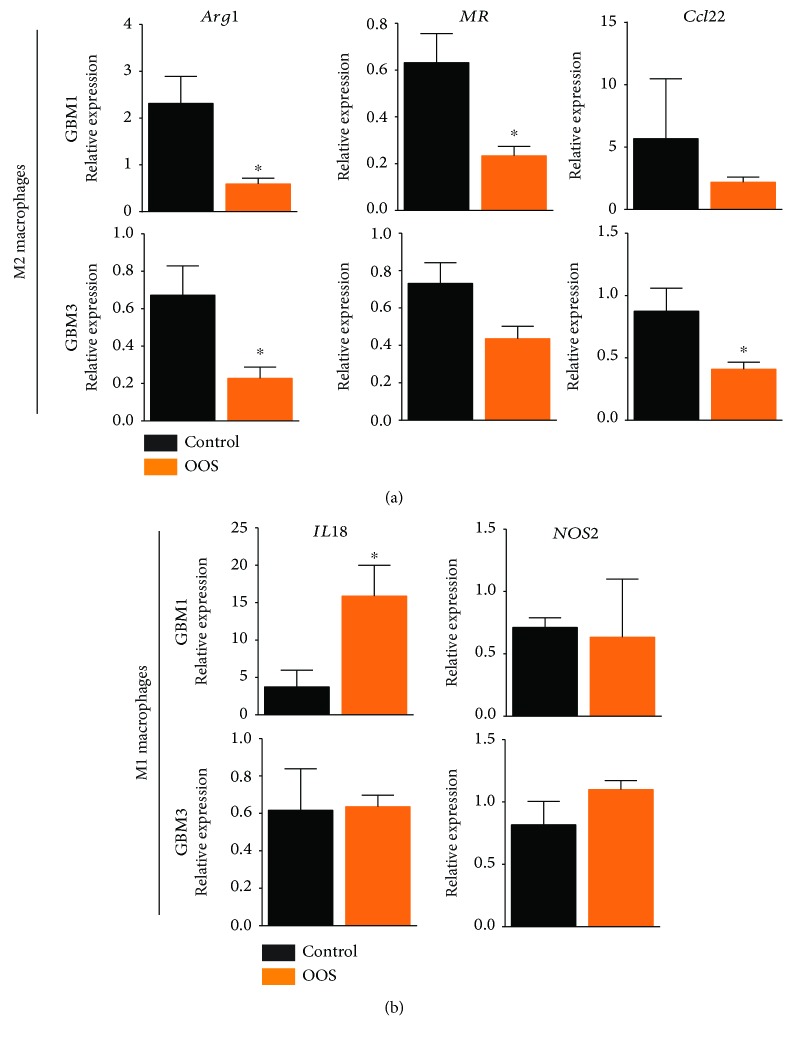
OOS affects macrophage polarization *in vivo*. GBM1 and GBM3 flank tumors were dissociated and the expression of different M2 (a) or M1 (b) markers was determined by qRT-PCR (*n* = 6). Mouse *Actin* was used for normalization. ^∗^ *P* ≤ 0.05.

Other authors have suggested that OOS increases the level of inflammatory cytokines [15]. To check if OOS has an effect on the immune component of GBMs, we performed a qRT-PCR analysis of a panel of markers of mouse lymphoid and myeloid cells in the dissected GBM1 and GBM3 tumors. We detected a change in macrophage polarization markers after OOS treatment, with a significant decrease in the expression of M2 (immunosuppressive) genes in GBM1 tumors as well as in GBM3 tumors (Figure 4(a)). We also detected an increase in some M1 (inflammatory) genes after OOS treatment, but only in GBM1 tumors (Figure 4(b)). GBM can contain large amounts of microglia and tumor-infiltrating macrophages, and their density is positively correlated with glioma grade, which suggests that they support tumor progression [28]. In fact, pharmacological or genetic inhibition of macrophage recruitment and M2 polarization blocks glioma growth [29]. Glioma-associated macrophages have been associated with proangiogenic, proinvasive and immunosuppressive functions, similar to those of alternatively activated M2 macrophages [30].

**Figure 5:**
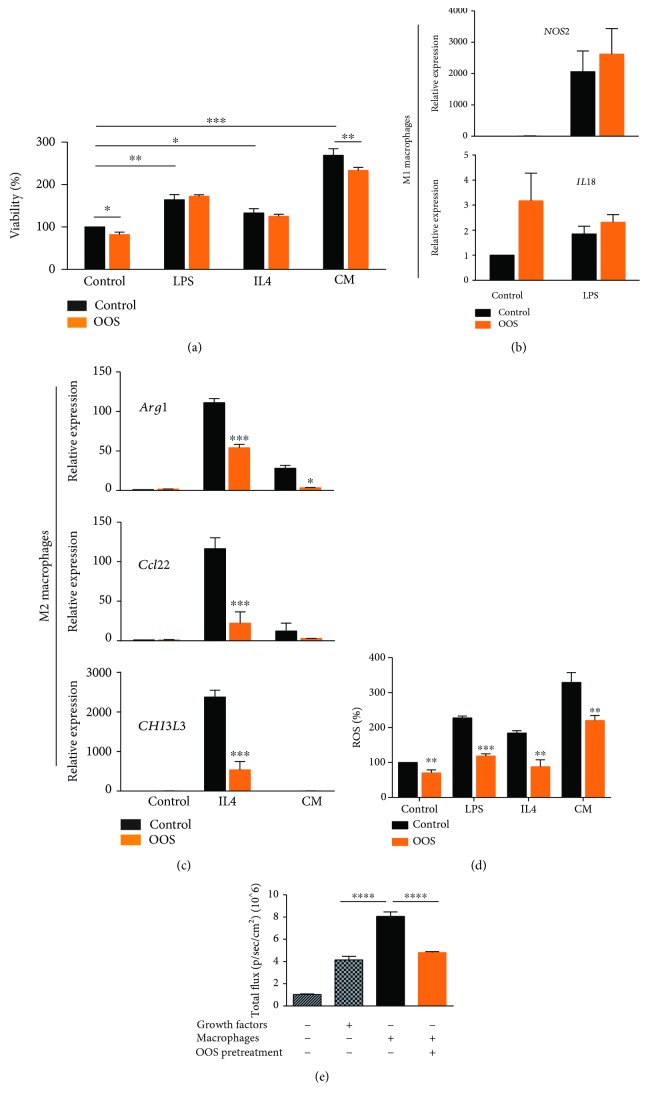
OOS affects macrophage polarization *in vitro*. (a) Cell viability was measured in the presence of the different stimuli. (b, c) Macrophage pellets were processed for qRT-PCR analysis of M1 (b) and M2 (c) genes. Mouse *Actin* was used for normalization, *n* = 3. (d) Analysis of ROS levels with DHE reagent in macrophages treated with the different stimuli, with or without OOS, *n* = 5. (e) Peritoneal macrophages were incubated in the presence of IL4 and OOS. After washing the macrophages, GBM1 cells were dissociated and plated on top of the macrophages or in control plates (with or without growth factors). Three days later, luciferase activity was measured in an IVIS equipment, *n* = 3. ^∗^ *P* ≤ 0.05; ^∗∗^ *P* ≤ 0.01; ^∗∗∗^ *P* ≤ 0.001; ^∗∗∗∗^ *P* ≤ 0.0001.

In order to decipher if OOS has a direct effect on macrophages, we cultured peritoneal macrophages in the presence and in the absence of the supplement. To induce M1 or M2 polarization, we used LPS (lipopolysaccharide) and IL4 (interleukin 4), respectively. We also incubated the macrophages in the presence of glioma-conditioned media (CM) (from GBM1). It has been shown that glioma cells release several factors that recruit and promote the growth of macrophages [28, 31]. We first determined that the viability of the macrophages was only slightly affected by the presence of OOS, although there was a clear stimulation in the presence of glioma-CM (Figure 5(a)), which was not able to induce M1 differentiation (data not shown). OOS did not significantly change the expression of M1 markers (in the absence or in the presence of LPS) (Figure 5(b)), although a small increment was observed in control-treated cells (*P* = 0.12). However, OOS clearly inhibited the upregulation of M2 markers induced by IL4 incubation and, to a lesser extent, by glioma-CM (Figure 5(c)), suggesting that OOS has a direct effect on these tumor inflammatory cells. Moreover, we observed that OOS was able to reduce the levels of ROS in the macrophages, independently of the differentiation stimuli (Figure 5(d)).

Macrophages that infiltrate glioma tissues are closely involved in the development of the tumor microenvironment by inducing angiogenesis, immunosuppression and invasion [28]. Moreover, it has been suggested that they can promote directly tumor cell proliferation. In order to analyze if the changes in macrophage polarization induced by OOS could affect secondarily glioma cells, we incubated GBM1 cells on top of macrophages that had been previously polarized by IL4, in the presence or in the absence of OOS. Results in Figure 5(e) suggest that there is indeed a protumoral function of M2 macrophages on glioma cells, as they support GBM1 growth even in the absence of growth factors. More importantly, pretreatment with OOS severely impaired this growth induction.

Altogether, these data reinforce the antitumor potential of OOS, as it could target CSCs and their supportive immunosuppressive microenvironment. However, in the xenograft setting, this general change in M2 polarization does not seem to be enough to inhibit tumor growth as OOS had no effect on GBM3 tumors (Figure 2), even though M2 markers were diminished (Figure 4(a)).

## 4. Discussion

In this study, we have evaluated the antitumor action of OOS in GBM using several human primary cell lines. OOS was able to reduce the *in vivo* growth of some of the GBM lines tested. We did not detect any changes in proliferation or cell death in the most sensitive tumors (GBM1 cells) (Supplementary Figure S1), suggesting that OOS does not have an effect on the overall tumor cell viability, as we had observed in the *in vitro* assays. However, there was a significant decrease in stem cell markers (*NESTIN* or CD44 expression) in response to OOS *in vitro* and *in vivo*. Moreover, the inhibition of the self-renewal capacity of GBM cells further confirmed that OOS inhibits the stem cell properties. Therefore, we cannot discard that OOS could be affecting proliferation or survival in the GBM CSC population.

Glioma CSCs are enriched in areas of high oxidative stress [4, 5]. Paradoxically, it has been suggested that low levels of ROS are required for stem cells to maintain quiescence and self-renewal, both in normal tissues and tumors [32]. In gliomas, CSCs appear to generate less ROS and have a higher ROS-scavenging capacity than more differentiated tumor cells [33]. In fact, an increase in mitochondrial ROS has also been linked to the loss of stem cell markers [34]. In contrast, our results indicate that the main effect of the antioxidant OOS is a decrease in the stemness properties of GBM. This observation agrees with recent results obtained with several ROS-scavenging compounds [7, 8]. Based on these data, it could be hypothesized that certain levels of ROS may be necessary to maintain CSCs in GBM cells, although too much oxidative stress could be also detrimental. Antioxidants could, therefore, deregulate this oxidative stress equilibrium, affecting stem cell properties and tumor growth.

Not all GBM CSCs seem to be sensitive to OOS. The supplement did not inhibit the clonal growth of GBM3 cells and it did not slow down the growth of GBM3 tumors, suggesting that CSCs from GBM3 are less sensitive to OOS than those from GBM1 or GBM2. It is important to remark that OOS was able to induce the expression of several antioxidant enzymes, but only in GBM1 and GBM2 cells. Moreover, the expression of some metabolic enzymes was very different in GBM3 cells compared to the other two lines, suggesting that the redox and the metabolic status of the glioma cells may determine their response to OOS. It would be interesting to test, in a larger cohort study, if the expression of some of these enzymes could be used as a predictive marker for the efficacy OOS or other antioxidant compounds.

Apart from the direct effect of OOS in GBM tumor cells, our data reflect that there was a change in the inflammatory component of the tumors, with a weaker expression of M2 macrophage markers in tumors treated with OOS. Glioma cells in general, and CSCs in particular, release several factors that recruit and promote the growth of macrophages [28, 31]. Therefore, changes in the properties of CSCs induced by OOS could be affecting the surrounding myeloid cells. Although we cannot discard such indirect effect, our results *in vitro* clearly indicate that OOS affects macrophage polarization in a direct way, reducing the expression of M2 markers in response to a classical inducer like IL4 but also in response to glioma-CM. We also confirmed that OOS reduces the levels of ROS in the macrophages in all the conditions tested. Although ROS production is usually associated with the activation and function of M1 macrophages [28], it has been recently shown that ROS are important in M2 but not in M1 macrophage differentiation. Thus, antioxidants like BHA (butylated hydroxy-anisole) [35], dihydroxycoumarins [36], or caffeic acid [37] inhibit M2 but not M1 polarization and prevent tumor growth and metastasis formation. Furthermore, chlorogenic acid, a product with antibacterial and antioxidant properties, inhibits the growth of GBM through the repolarization of macrophage from M2 to M1 phenotype [38]. Therefore, OOS could exert their antitumorigenic action, at least in part, through the inhibition of M2 differentiation. Other authors have observed an increase in proinflammatory cytokines like IL6 in mouse models of acute leukemia treated with OOS [15], suggesting that the macrophage-polarization effect of OOS could be extended to other cancers. In our hands, however, OOS does not induce a significant increase in the expression of M1 markers in LPS-treated macrophages, although *IL18* (but not *NOS2*) is overexpressed in GBM1 tumors treated with OOS. IL18 is another cytokine secreted by M1 macrophages that participates in the activation of T cell-mediated inflammatory responses [39]. Interestingly, in GBM3 tumors, M2 markers are also inhibited whereas *IL8* expression is not induced in response to OOS. This could participate in the lack of response of these cells to the dietary supplement. Moreover, these results suggest that other components of the glioma (including the tumor cells) might modulate the response of macrophages to OOS, making some tumors more susceptible to these changes than others.

It is important to remark that, in our hands, treatment with OOS does not have any apparent toxic effect since no differences were observed in animal weight or behavior in control and treated mice. Some oncologists avoid the use of antioxidant supplements during treatment because it has been reported that they may have detrimental effects or even undesirable interactions with certain therapies. However, several articles have concluded that antioxidant supplements do not undermine the effectiveness of cytotoxic therapies [10]. Therefore, it would be interesting to find out if there is a synergy between OOS and the conventional chemotherapy (TMZ), as it has been shown in other types of cancer [15, 40]. The fact that OOS has been marketed for years without any report of adverse effects would help in verifying this synergism in the clinic. Moreover, it would be interesting to test whether OOS could have a similar beneficial effect in combination with radiotherapy or even for the novel immunotherapies that are currently in early clinical phases.

## 5. Conclusions

- The presence of Ocoxin® oral solution (OOS) inhibits the self-renewal capacity of a percentage of primary glioblastoma (GBM) cell lines
- Systemic treatment with OOS reduces tumor burden of a percentage of primary GBMs
- OOS inhibits M2 protumoral polarization of macrophages *in vitro*
- Systemic treatment with OOS inhibits the M2 protumoral polarization of glioma-associated macrophages
- OOS is a feasible candidate to be used in combination therapies during the treatment of GBM patients

## Data Availability

The mouse models and the molecular and cellular data used to support the findings of this study are included within the article and within the supplementary information files.

## Supplementary Materials

Supplementary Materials Supplementary Figure S1: full-length blots of Figure 1(g). GBM cells were treated with OOS (1 : 100 or 1 : 50) for 2 or 24 h and expression of Nrf-2 and NQO-1, and HO-1 was measured by WB. Nontreated spheres were used as a control and the expression of GAPDH as a loading control. Supplementary Figure S2: OOS does not induce overall changes in proliferation or cell death in GBM1 tumors. Paraffin sections of GBM1 flank tumors were stained with hematoxylin-eosin to count the number of mitosis (upper panel) or were stained with Activated Caspase 3 antibody to count apoptotic cells (lower panel). In both cases, the graphs represent the number of cells counted in 10 fields per tumor (*n* = 10). Supplementary Table S1: list of primary antibodies for immunohistochemical DAB staining (IHC), flow cytometry (FC) and western blot (WB). Supplementary Table S2: list of secondary antibodies for immunohistochemical DAB staining (IHC) and western blot (WB). Supplementary Table S3: list of primers used for RT-PCR.
