# Supplementary material for: Ocoxin Modulates Cancer Stem Cells and M2 Macrophage Polarization in Glioblastoma
Source: Oxid Med Cell Longev. 2019 Aug 5;2019:9719730. doi: 10.1155/2019/9719730 (PMC6701394; doi:10.1155/2019/9719730)
Supplement: Supplementary Materials — Supplementary Figure S1: full-length blots of Figure 1(g). GBM cells were treated with OOS (1 : 100 or 1 : 50) for 2 or 24 h and expression of Nrf-2 and NQO-1, and HO-1 was measured by WB. Nontreated spheres were used as a control and the expression of GAPDH as a loading control. Supplementary Figure S2: OOS does not induce overall changes in proliferation or cell death in GBM1 tumors. Paraffin sections of GBM1 flank tumors were stained with hematoxylin-eosin to count the number of mitosis (upper panel) or were stained with Activated Caspase 3 antibody to count apoptotic cells (lower panel). In both cases, the graphs represent the number of cells counted in 10 fields per tumor (n = 10). Supplementary Table S1: list of primary antibodies for immunohistochemical DAB staining (IHC), flow cytometry (FC) and western blot (WB). Supplementary Table S2: list of secondary antibodies for immunohistochemical DAB staining (IHC) and western blot (WB). Supplementary Table S3: list of primers used for RT-PCR. [file 9719730.f1.pdf]

Title: Ocoxin modulates cancer stem cells and M2-macrophage polarization in glioblastoma.

Authors: Esther Hernández-SanMiguel[1], Ricardo Gargini[2], Teresa Cejalvo[3], Berta Segura-Collar[1], Paula Núñez-Hervada[1], Rafael Hortigüela[1], Juan M. Sepúlveda-Sánchez[3], Aurelio Hernández-Laín[3], Angel Pérez-Núñez[3], Eduardo Sanz[4], Pilar Sánchez-Gómez[1]

1 Neurooncology Unit, Instituto de Salud Carlos III-UFIEC, Madrid, Spain

2 Centro de Biología Molecular, CSIC, Madrid, Spain

3 Unidad Multidisciplinar de Neurooncología, Hospital Universitario 12 de Octubre, Madrid, Spain

4 Catalysis S.L., Madrid, Spain

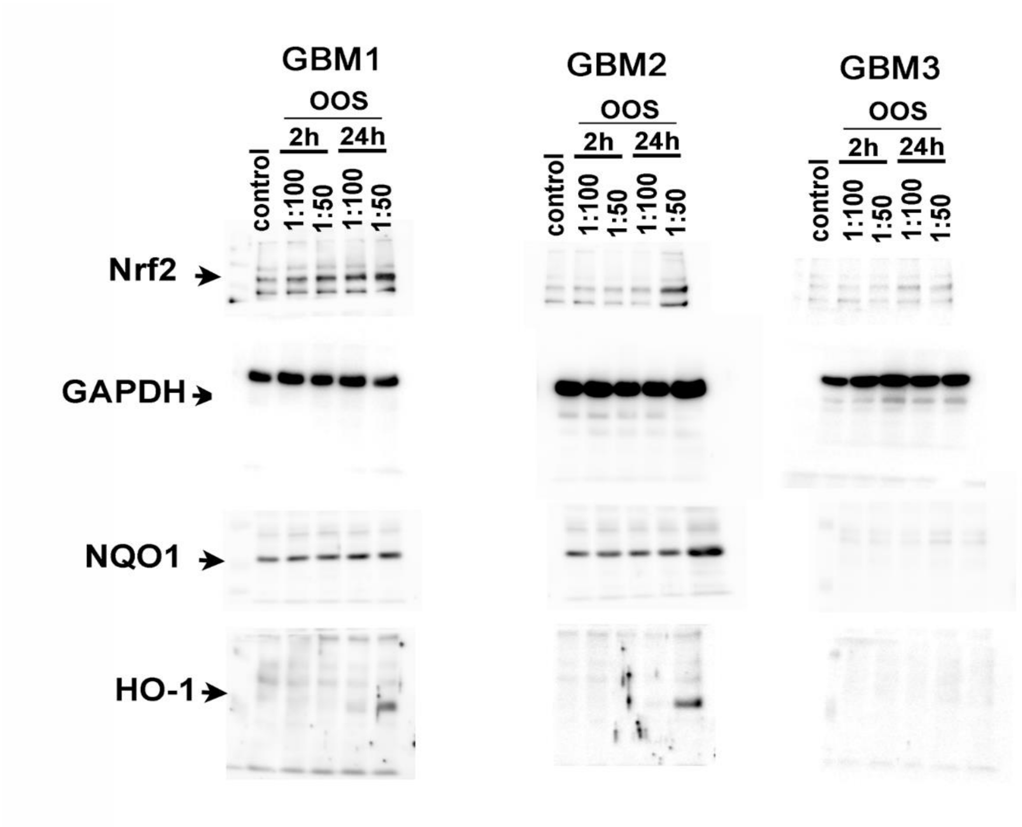

Supplementary Figure S1. Full-length blots of Figure 1E. GBM cells were treated with OOS (1:100 or 1:50) for 2 or 24h and expression of Nrf2, NQO1 and HO-1 was measured by WB. Non-treated spheres were used as a control and the expression of GAPDH as a loading control.

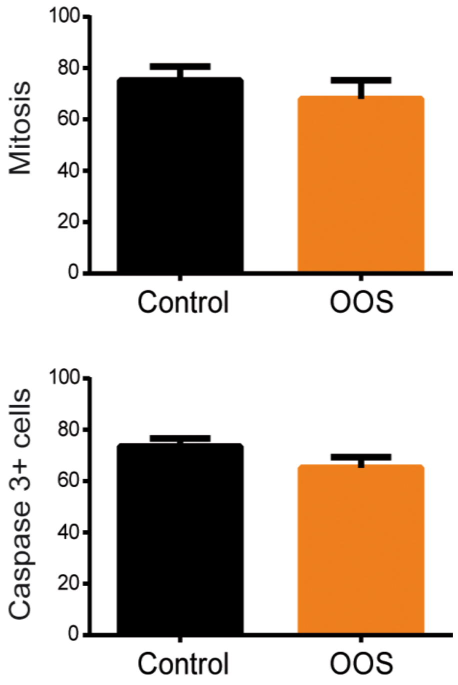

Supplementary Figure S2. OOS does not induce overall changes in proliferation or cell death in GBM1 tumors. Paraffin sections of GBM1 flank tumors were stained with hematoxylin-eosin to count the number of mitosis (upper panel) or were stained with Activated Caspase 3 antibody, to count apoptotic cells (lower panel). In both cases, the graphs represent the number of cells counted in 10 fields per tumor (n=10).

Primary antibody	Host species	Use dilution	Provider
**Activated Caspase-3**	Rabbit	1:100 (IHC)	Cell Signaling
**CD44-FITC**	Mouse	1:10 (FC)	ImmunoTools
**GAPDH**	Mouse	1:1000 (WB)	Santa Cruz
**HO-1**	Rabbit	1:1000 (WB)	Millipore
**NQO-1**	Goat	1:2000 (WB)	Abcam
**Nrf2**	Rabbit	1:1000 (WB)	Diagenode

Supplementary Table S1: List of primary antibodies for immunohistochemical DAB staining (IHC), flow cytometry (FC) and western blot (WB).

Secondary antibody	Host species	Use dilution	Provider
**Biotinylated anti rabbit**	horse	1:200 (IHC)	Vector
**HRP-conjugated anti goat**	rabbit	1:2000 (WB)	Dako
**HRP-conjugated anti mouse**	sheep	1:5000 (WB)	GE Healthcare
**HRP-conjugated anti rabbit**	donkey	1:5000 (WB)	GE Healthcare

Supplementary Table S2: List of secondary antibodies for immunohistochemical DAB staining (IHC) and western blot (WB).

Gene name	Forward primer 5’-3’	Reverse primer 5’-3’
hACSS1	GGTGGACTCCATGCCTCTT	AGAGCGTTGCTTTGATCTGG
hCAT	TTAATCCATTCGATCTCACC	GGCGGTGAGTGTCAGGATAG
hGSR	TGGCACTTGCGTGAATGTTG	CACATAGGCATCCCGCTTTTC
hLDHC1	CTTGCACCTGCTGTGACAAT	TTCAGCATGGCAGTCTTTTC
hNESTIN	GCGGCTGCGGGCTACTGAAA	CCAGCTGCTGCCGACCTTCC
hCD133	GCCACCGCTCTAGATACTGC	TGTTGTGATGGGCTTGTCAT
hCD44	AGAAGGTGTGGGCAGAAGAA	AAATGCACCATTTCCTGAGA
hSOX2	GCGAACCATCTCTGTGGTC	AATGGAAAGTTGGGATCGAA
hSOD2	GGCCTACGTGAACAACCTGAA	CTGTAACATCTCCCTTGGCCA
hTUB	TGGAACCCACAGTCATTGATGA	TGATCTCCTTGCCAATGGTGTA
bmActin	CCGGGACCTGACAGACTACCT	GCCATCTCCTGCTCGAAGTCTA
mArg1	TGAGAGACCACGGGGACCTG	GCACCACACTGACTCTTCCATTC
mCcl22	CATCATGGCTACCCTGCGTGTCCC	CCTCCTCCCTAGGACAGTTTATGGA
mCHI3L3	TGGAATTGGTGCCCCTACAA	AACTTGCACTGTGTATATTG
mIL18	CATGTACAAAGACAGTGAAGTAAGAGG	TTTCAGGTGGATCCATTTCC
mMR	GTAGTACCGGAGGGTGCAGA	TTTGCATCAGTGAAGGTGGA
mNOS2	GAGCTGGGCTGTACAAACCTT	CATTGGAAGTGAAGCGTTTCG
h-mRPII	GCACCACGTCCAATGACAT	GTGCGGCTGCTTCCATAA

Supplementary Table S3: List of primers used for RT-PCR.
